# Prickly Pear and Pomegranate Peel Extracts as Natural Antioxidants: Effects on Colour, Lipid, and Protein Oxidation in Refrigerated Cooked Chicken Models

**DOI:** 10.3390/foods14091568

**Published:** 2025-04-29

**Authors:** Guadalupe Lavado, Ramón Cava

**Affiliations:** TRADINNOVAL Research Group, INBIO G+C, Universidad de Extremadura, 10003 Cáceres, Spain; guadalupelr@unex.es

**Keywords:** pomegranate, prickly pear, peel extracts, protein oxidation, lipid oxidation, cooked products, colour

## Abstract

This study investigates the antioxidative potential of pomegranate peel extract (PPE) and prickly pear peel extract (HPE) as natural preservatives in cooked chicken models. The extracts were characterized for their phenolic and tannin content, and their antioxidant activity was measured through in vitro chemical assays using ABTS, DPPH, and FRAP assays. Cooked chicken samples were formulated with different concentrations of PPE or HPE and compared to sodium nitrite (NaNO_2_) treatment. The effects on lipid and protein oxidation, instrumental colour parameters, and aldehyde formation were evaluated during storage. The results demonstrated that PPE exhibited higher antioxidant activity compared to HPE, particularly at higher concentrations. PPE_300 had the highest phenolic content, exhibited the strongest radical scavenging activity, and significantly reduced lipid oxidation markers such as malondialdehyde and lipid hydroperoxides. PPE also preserved protein integrity by reducing carbonyl formation and maintaining thiol levels. Colour stability was improved in both PPE- and HPE-treated samples, although nitrite remained the most effective in maintaining redness (a*-values). These findings suggest that PPE, particularly at 300 mg/kg, is a promising natural alternative to synthetic antioxidants for improving oxidative stability and shelf life in meat products. Further research should explore sensory attributes and consumer acceptance to facilitate industrial applications.

## 1. Introduction

Food safety, sensory attributes, and shelf life are critical concerns for food scientists, as they directly impact consumer health and product quality [1]. Sodium nitrite has long been used in meat products due to its antimicrobial and antioxidant effects—it inhibits pathogens such as *Clostridium botulinum*, *Listeria*, and *Salmonella*, prevents oxidation, and enhances flavour and colour [2,3].

Despite its functional benefits, the use of nitrite in food has raised increasing health concerns among consumers and researchers alike. While nitrites play a vital role in maintaining microbial safety, colour, and oxidative stability in processed meats, their potential to form carcinogenic nitrosamines has led to a shift in consumer preferences toward cleaner-label, additive-free alternatives [4]. This demand poses a significant challenge for the food industry: to maintain product safety and sensory quality while reducing or eliminating the use of nitrites [5].

In response to this challenge, there has been growing interest in the use of natural antioxidants derived from plant sources—such as spices, fruits, and their by-products—as viable alternatives to synthetic additives [1,6,7,8]. Among these, pomegranate (*Punica granatum* L.) and prickly pear (*Opuntia ficus-indica* L.) have emerged as promising candidates due to their high content of bioactive polyphenols, including flavonoids, phenolic acids, and tannins [9,10,11,12].

This study aims to evaluate the effectiveness of pomegranate peel extract (PPE) and prickly pear peel extract (HPE) as natural antioxidants in nitrite-free cooked chicken models. Specifically, we hypothesize that (1) both extracts will contribute to oxidative stability during refrigerated storage; (2) the antioxidant effect will depend on the source; and (3) their efficacy will be dose-dependent.

Pomegranate peel is known for its rich content of ellagitannins (e.g., punicalagin), flavonols (e.g., quercetin), and anthocyanins, which contribute not only to antioxidant activity but also to pigmentation [13]. Thus, anthocyanins provide it with a red hue, while ellagitannins like punicalagin and ellagic acid produce reddish-brown tones. Additionally, flavonols such as quercetin and luteolin, along with small amounts of carotenoids, further enhance the pigmentation [11]. Numerous studies have demonstrated the efficacy of pomegranate extracts, powders, and juices in improving oxidative stability in various meat matrices, including pork, beef, chicken, and lamb, as well as in processed products such as sausages and burgers [10,14,15,16,17]. Specifically, PPE has shown effectiveness in delaying lipid and protein oxidation, thus extending shelf life when used as a partial or full substitute for nitrites in cooked [18,19] and dry sausages [20,21].

While the functional potential of pomegranate is well established, prickly pear remains an underexplored source of natural antioxidants, despite its composition of diverse phenolic compounds such as flavonols, stilbenes, and betalain pigments [22]. The characteristic coloration of prickly pear fruit, primarily due to betanin (red-purple) and indicaxanthin (yellow-orange), indicates the presence of bioactive compounds with antioxidant properties [23]. Recent studies have begun to demonstrate the feasibility of using prickly pear in meat systems, with positive results in colour stabilization and inhibition of lipid oxidation in products such as chicken patties and burgers [9,24]. However, comparative studies assessing the antioxidant potential of prickly pear against more established matrices like pomegranate are limited.

Therefore, this study represents a first step in comparing two natural extracts—one well-established (pomegranate) and one emerging (prickly pear)—to assess their capacity to replace nitrites in cooked chicken products. Both extracts were tested at two concentrations (30 and 300 mg total phenolic compounds per kg of meat), and their effects were evaluated in terms of instrumental colour parameters and oxidative stability markers during refrigerated storage.

Lipid oxidation was assessed through the quantification of lipid hydroperoxides (LOOH), malondialdehyde (MDA), 4-hydroxynonenal (4-HNE), and fatty aldehydes (pentanal, hexanal, heptanal, octanal, and nonanal). Protein oxidation was evaluated using markers such as protein carbonyls, tryptophan loss, dityrosine formation, Schiff base fluorescence, and thiol content. The findings from this study aim to inform future efforts in developing clean-label meat products and provide a scientific basis for scaling these applications to an industrial level.

## 2. Materials and Methods

### 2.1. Chemicals and Equipment

The chemical reagents were sourced from Fisher Scientific Co. (Waltham, MA, USA), while pentanal, hexanal, heptanal, octanal, and nonanal were purchased from Merck KGaA (Darmstadt, Germany). The 4-hydroxynonenal standard (4-HNE), in its 4-HNE DMA form, was supplied by Avanti Polar Lipids (Alabaster, AL, USA).

The assays were conducted at controlled temperatures using a Thermomixer C (Eppendorf AG, Hamburg, Germany), equipped with a heated lid. Sample transfers to 96-well microplates were performed with an Epmotion 5073 liquid handling workstation (Eppendorf, Hamburg, Germany) prior to spectrophotometric or fluorometric measurements. Absorbance and fluorescence readings were recorded using the Varioskan LUX Multimode Microplate Reader (Thermo Fisher Scientific, Vantaa, Finland). All assays were performed in duplicate, with each reading taken in quadruplicate.

High-performance liquid chromatography (HPLC) analysis was carried out using a Shimadzu SIL 20A HT system, coupled with a CTO 20AC column oven and an RF 20A XS fluorescence detector (Shimadzu Co., Kyoto, Japan).

### 2.2. Preparation of Pomegranate Peel Extract (PPE) and Prickly Pear Peel Extract (HPE)

Pomegranate (*Punica granatum* L.) fruits (n = 20, cultivar “Mollar”) and prickly pear (*Opuntia ficus-indica* L.) fruits (n = 50, “verdal” cultivar) were harvested directly from pomegranate trees and prickly pear cactus, respectively, during the autumn season (Cáceres, Spain).

The pomegranates and prickly pear fruits were washed, and then peels were removed. Peels were cut into 1 cm^2^ pieces and dried for 24 h at 60 °C in an oven. Once dried, peels were ground using a GM 200 blade mill (Retsch GmbH, Haan, Germany).

Chemical analysis of the normalized HPE and PPE extracts at 30 and 300 mg TPC/mL revealed differences. The extraction was conducted at a 1:9 (*w*/*v*) peel-to-solvent ratio for 2 h at 60 rpm in the dark with a rock&roller tube shaker (Cole-Parmer, Staffordshire, UK). Following extraction, the mixture was centrifuged at 5000 rpm for 5 min, filtered, and stored at −70 °C. Prior to use, acetone was removed by rotary evaporation at 40 °C using a Hei Vap Advantage rotary evaporator (Heidolph GmbH, Schwabach, Germany).

Both extracts were standardized to 30 and 300 mg of total phenolic compounds/mL for use in cooked chicken models at 30 and 300 mg TPC/kg. The selected doses were based on expressing antioxidant addition levels in mg of total phenolic content (TPC) per kg, rather than as extract percentages, to enable consistent comparisons across different plant extracts by standardizing the active phenolic compound content despite compositional variability.

### 2.3. Quantification of Antioxidant Compounds

#### 2.3.1. Total Phenolic Content (TPC)

Total phenolic content was measured using the Folin–Ciocalteau method [25]. In a test tube, 50 μL of extract, 450 μL of MilliQ water, and 20 μL of Folin–Ciocalteau reagent were vortexed. Then, 50 μL of 20% Na_2_CO_3_ and 450 μL of MilliQ water were added. After incubating for 1 h at 25 °C, the solution was transferred to a 96-well plate, and absorbance was measured at 765 nm using a Varioskan Lux plate reader (Thermo Fisher Scientific, MA, USA). A gallic acid calibration curve (10–500 μg/mL) was used, and results were expressed as mg gallic acid equivalents (GAE) per gram of sample. Assays were performed in duplicate.

#### 2.3.2. Condensed Tannin Content

Condensed tannins were quantified using the method of Waterman and Mole [26]. In a 96-well plate, 25 μL of extract and 250 μL of vanillin reagent (1% vanillin in MeOH with 10% HCl) were added to each well and incubated for 30 min. Absorbance was measured at 500 nm using a plate reader. A (+)-catechin calibration curve (0.16–10 mg/mL) was used, and results were expressed as mg catechin per gram of sample. Assays were performed in duplicate.

### 2.4. Antioxidant Activity Assays

#### 2.4.1. ABTS Assay

The ABTS radicals (ABTS^•+^) were generated by mixing a 7 mM ABTS solution with 2.45 mM K_2_S_2_O_8_ in a 1:1 ratio, and kept in the dark for 16 h [27]. The solution was diluted with ethanol to achieve an absorbance of 0.7 ± 0.1 at 734 nm. In a 96-well plate, 5 μL of extract and 245 μL of ABTS^•+^ solution were combined, and absorbance was measured at 734 nm for 7 min at 60-s intervals. A Trolox calibration curve (0.25–1.0 mg/mL) was used, and results were expressed as mg Trolox equivalents (TEAC) per mL of extract. Assays were conducted in quadruplicate.

#### 2.4.2. DPPH Radical Scavenging Assay

The DPPH• solution (100 μM) was prepared in 80% methanol, and absorbance was measured at 515 nm [28]. In a 96-well plate, 10 μL of extract (1:100 dilution) and 290 μL of DPPH• solution were mixed. After 30 min of incubation, absorbance was recorded at 515 nm. Radical inhibition was calculated using the formula:

% Inhibition at 515 nm = [1 − (A_f_/A_0_)] × 100

where A_0_ is the DPPH absorbance without extract, and A_f_ is the absorbance after extract addition. Assays were performed in quadruplicate.

#### 2.4.3. FRAP Assay

The FRAP reagent was prepared by mixing 2.5 mL of 10 mM TPTZ, 2.5 mL of 20 mM FeCl_3_, and 25 mL of 0.3 mM acetate buffer (pH 3) [28]. In a 96-well plate, 200 μL of FRAP reagent was added to each well, followed by 7 μL of extract and 20 μL of water. The plate was incubated for 30 min at 25 °C, and absorbance was recorded at 593 nm. A Trolox calibration curve (0.25–4.0 mg/mL) was used, and the results were expressed as TEAC (Trolox equivalents antioxidant capacity) per millilitre of extract. Assays were conducted in quadruplicate.

### 2.5. Preparation of Cooked Chicken Models

Commercial chicken breasts were obtained from a local meat market. The formulation for the preparation of the cooked chicken models is shown in Table 1. Chicken breast (moisture: 76.1 ± 0.48, protein: 21.1 ± 0.53, fat: 2.1 ± 0.45, collagen: 0.6 ± 0.14, expressed as g/100 g) was minced using a meat grinder equipped with a 4-mm-diameter plate (Mainca, Barcelona, Spain). Minced chicken breast, water, salt, nitrite, and extracts were then chopped and mixed in a Universal Machine UMC 5–12 (Stephan Machinery GmbH, Hameln, Germany) at 300 rpm, at 4 °C for 5 min.

Following mixing, batters (~50 g) were transferred into 50 mL polypropylene tubes and cooked in a water bath until reaching an internal temperature of 70 °C, which was maintained for 30 min. The core temperature was monitored using a K-type thermocouple sensor connected to a data logger (Data Harvest, Bedfordshire, UK). The cooked products were cooled in cold water for 10 min. To assess changes during refrigerated storage, samples were kept in polypropylene plastic tubes under aerobic conditions and stored at +4 °C for 10 d in the dark.

Six experimental groups were established based on the inclusion of sodium nitrite (NaNO_2_) and pomegranate peel extract (PPE) or prickly pear peel extract (HPE) in the formulations, as detailed below:

a. 0 mg/kg NaNO_2_ and 0% PPE (0_Nitr).
b. 150 mg/kg NaNO_2_ and 0% PPE (150_Nitr).
c. 0 mg/kg NaNO_2_ and 30 mg of phenolic compounds/kg from PPE (30_PPE).
d. 0 mg/kg NaNO_2_ and 300 mg of phenolic compounds/kg from PPE (300_PPE).
e. 0 mg/kg NaNO_2_ and 30 mg of phenolic compounds/kg from HPE (30_HPE).
f. 0 mg/kg NaNO_2_ and 300 mg of phenolic compounds/kg from HPE (300_HPE).

### 2.6. Instrumental Colour, Lipid and Protein Oxidation Markers

#### 2.6.1. Instrumental Colour Parameters

Colour measurements were performed according to AMSA [29]. Instrumental colour coordinates CIE L*a*b* were measured using a CM-700d portable spectrophotometer (Minolta Camera, Osaka, Japan). The device was set to illuminant D65, 10º standard observer, and 8 mm port/viewing area. Prior to colour measurement, the colorimeter was standardized with a white (CR-A43) and a black tile (CM-A182). Three colour readings at three randomly selected places were taken from each sample, and the average value was used. All measurements were carried out on transversally cut sections of the CCPs at 0, 5, and 10 days of refrigerated storage. The total colour changes (Δ*E*) were calculated to compare differences between control, nitrite, PPE, and HPE treatments (Equation (1)):

(1) $$∆E^{*}=\sqrt{{\left(L^{*}-L_{ref}^{*}\right)}^{2}+{\left(a^{*}-a_{ref}^{*}\right)}^{2}+{\left(b^{*}-b_{ref}^{*}\right)}^{2}}$$

where L*_ref_, a*_ref_, and b*_ref_, represents the L*, and a* and b* values in the control or nitrite group.

#### 2.6.2. Quantification of Lipid Hydroperoxides

Lipid hydroperoxide levels were determined according to Mihaljević et al. [30]. A 0.7 g sample of uncured dry sausage was homogenized in 7 mL of distilled water at 15,000 rpm for 30 s. To 500 µL of homogenate, 500 µL of 0.0834 M (HPO_3_)n in MeOH was added, followed by 1 mL of chloroform. The mixture was vortexed, centrifuged at 1500× *g* for 5 min at 0 °C, and the organic phase (500 µL) was mixed with 450 µL of chloroform/MeOH (2:1). After adding 50 µL of 4.5 mM ferrous sulphate: 3% ammonium thiocyanate (1:1), the mixture was stirred for 5 min at 22 °C. Absorbance was measured at 500 nm. A standard curve of 13-hydroperoxy octadecadienoic acid (5–0.5 nmol) was used for quantification, with results expressed as nmol/g of sample.

#### 2.6.3. Quantification of Malondialdehyde (MDA)

MDA was quantified according to Lavado et al. [28]. A 0.7 g sample was homogenized in 7 mL of distilled water. A 150 µL aliquot was mixed with 50 µL of 3N NaOH and heated at 60 °C for 30 min. Then, 250 µL of 6% H_3_PO_4_ and 250 µL of 0.8% thiobarbituric acid were added, and the mixture was heated at 90 °C for 45 min. After cooling, MDA-TBA adducts were extracted with 100 µL of 10% SDS and 600 µL of butanol, followed by centrifugation at 3000× *g* for 10 min. Absorbance of the butanol layer was measured at 532 nm. A standard curve of 1,1,3,3-tetramethoxypropane (7.2–0.056 µg MDA/mL) was used, with results expressed as µmol/g of sample.

#### 2.6.4. Quantification of 4-Hydroxynonenal (4-HNE) and Saturated Aldehydes

The 4-HNE and aldehydes were quantified by HPLC-FD after derivatization with 1,3-cyclohexanedione (CHD) [28]. A 400 µL sample was mixed with 1 mL of CHD reagent and 100 µL of 80 µM BHT-80 µM EDTA in methanol, vortexed, and heated at 60 °C for 1 h with constant stirring. After cooling, 500 µL of methanol was added, and the mixture was centrifuged. The supernatant was filtered through a 0.2 µm PTFE filter. HPLC was performed using a Shimadzu system with a Cosmosil 5C18-MS-II column, applying a linear THF gradient. Detection was by fluorescence (excitation: 380 nm, emission: 446 nm). Standards for pentanal, hexanal, heptanal, octanal, nonanal, and 4-HNE were used for identification and quantification. Results were expressed as ng/g of sample.

#### 2.6.5. Quantification of Protein Carbonyls

Protein carbonyls were quantified according to Soiglia et al. [31]. A 1 g sample was homogenized in 10 mL of 0.15 M KCl solution at 9500 rpm for 30 s. Aliquots (100 µL) were mixed with 1 mL of 10% trichloroacetic acid (TCA) and centrifuged at 5000× *g* for 5 min. The pellet was resuspended in 400 µL of 5% SDS, heated at 100 °C for 10 min, and ultrasonicated at 40 °C for 30 min. The samples were incubated with 0.8 mL of 0.3% 2,4-dinitrophenylhydrazine (DNPH) in 3 M HCl, while blanks were treated with 3 M HCl. After precipitation with 40% TCA, the pellet was washed and dried. The pellet was dissolved in 1.5 mL of 6 M guanidine hydrochloride and incubated overnight at 4 °C. Absorbance at 280 nm and 370 nm was measured for protein concentration and carbonyl content, respectively. Results were expressed as nmol carbonyls/mg protein.

#### 2.6.6. Quantification of Thiols

Thiol concentrations were determined according to Ellman [32], with modifications [33]. A 1.5 g dry sausage sample was homogenized in 12.5 mL of 0.05 M MES buffer (pH 5.8) at 11,600 rpm for 30 s. A 250 µL aliquot was mixed with 750 µL of 5% sodium dodecyl sulphate (SDS) in 0.10 M TRIS buffer (pH 8.0) and incubated at 80 °C for 30 min. After centrifugation at 3000× *g* for 20 min, the supernatant was filtered through a 0.45 µm PTFE filter. Thiol concentration was determined by reacting 50 µL of the sample with 50 µL of 10 mM 5,5′-dithiobis(2-nitrobenzoic acid) (DTNB) in TRIS buffer, and absorbance was measured at 412 nm before and after 30 min of reaction. A standard curve of L-cysteine was used, and results were expressed as nmol Cys eq./mg protein.

#### 2.6.7. Tryptophan Fluorescence

The tryptophan fluorescence in the homogenate samples was measured using a fluorescence spectrometer [34]. Emission spectra were recorded in the range of 300 to 500 nm, with the excitation wavelength fixed at 350 nm. Emission spectra of extraction buffer, recorded under identical conditions, were used as the background. The excitation and emission slit widths were set to 10 nm, and data acquisition was performed at a rate of 500 nm per minute.

#### 2.6.8. Dityrosine Fluorescence Measurements

Dityrosine production was followed by fluorescence measurements on sample homogenates [35]. The excitation and emission wavelengths were set to 325 nm and 420 nm, with a slit width of 10 nm. Relative fluorescence values were denoted as relative fluorescence units (RFU).

#### 2.6.9. Schiff Base Fluorescence Spectroscopy

The emission of fluorescence by the Schiff base was assessed using a fluorescence spectrometer [34]. A 1 mL aliquot of the homogenates was redissolved in 20 mL of the 20 mM sodium phosphate buffer, and the emission spectra of the Schiff base were recorded from 400 to 500 nm, with the excitation wavelength set at 350 nm. Excitation and emission slit widths were set to 10 nm, and data were collected at 500 nm per minute. Results were expressed as fluorescence intensity units (RFU) emitted at 460 nm.

### 2.7. Statistical Analysis

The experiment was conducted using a completely randomized design. A total of 90 cooked chicken models were evaluated across various dependent variables, involving 6 treatments × 3 sampling points × 5 sample repetitions per sampling point. Data normality was assessed using the Kolmogorov–Smirnov test, and outliers were evaluated prior to statistical analysis. No data points were excluded. The data underwent a one-way analysis of variance (ANOVA) to assess stability, considering storage time and treatment as fixed effects. When significant differences were observed (*p* < 0.05), Tukey’s HSD post-hoc test was applied to compare means. A Pearson’s correlation analysis was computed to assess the relationship among variables. SPSS Statistics IBM SPSS Statistics V22.0 [36] was used to perform statistical analysis, and results are presented as means ± standard deviations.

## 3. Results

### 3.1. Chemical Characterization of Prickly Pear and Pomegranate Peel Extracts

The chemical profiles of prickly pear (HPE) and pomegranate peel extracts (PPE) revealed marked differences in their bioactive compound concentrations and antioxidant capacities (Table 2). 

The phenolic content of both prickly pear extract (HPE) and pomegranate peel extract (PPE) exhibited significant variations depending on the concentration used. As expected after extract preparation, HPE_300 and PPE_300 contained ten times more total phenolic compounds (320.6 and 328.9 mg/mL, respectively) than their HPE_30 and PPE_30 (31.5 mg/mL each) counterparts. Additionally, condensed tannins were exclusively detected in PPE_300 (1.2 ± 0.14 mg/kg), suggesting a distinct compositional advantage in terms of polyphenolic profile.

Antioxidant assays further supported these findings. For FRAP, PPE_300 exhibited the highest activity (555.8 µg Fe^2^⁺/mL), followed closely by HPE_300 (150.1 µg Fe^2^⁺/mL). Both extracts demonstrated dose-dependent enhancements in DPPH and ABTS radical-scavenging activities. PPE_300 achieved the highest values in both assays (DPPH: 627.5 mg Trolox eq./mL; ABTS: 807.8 mg Trolox eq./mL), reflecting its superior radical-quenching efficacy. These results confirm the extracts’ high antioxidative capacity, with PPE outperforming HPE, particularly at higher concentrations.

### 3.2. Instrumental Colour

The inclusion of nitrite, or varying concentrations of prickly pear (HPE) or pomegranate peel (PPE) extracts, significantly (*p* < 0.001) affected the CIEL*a*b* colour coordinates (Table 3). Refrigerated storage did not influence these changes resulting from the incorporation of nitrite, HPE, or PPE.

No significant differences in lightness (L*) were observed between the control group and the nitrite group. HPE_300, PPE_30, and PPE_300 significantly (*p* < 0.05) reduced L*-values, indicating darker samples than the control and nitrite groups, with higher doses causing a more marked darkening effect.

The CIE a*-values displayed distinct trends based on the cooked chicken formulation. The nitrite group exhibited the highest a*-value, indicating a more intense redness, while the control group showed the lowest a*-value. The addition of HPE and PPE resulted in significantly (*p* < 0.05) higher a*-values than the control samples, but these values were still significantly lower than those in the nitrite-treated samples. No significant dose-dependent effect was observed in the PPE group on the a*-values, suggesting that varying the PPE dose did not significantly affect the red-green balance of the samples. In contrast, a dose-dependent effect was observed for the HPE treatment, with higher doses resulting in significantly (*p* < 0.05) higher a*-values. Thus, the ranking of a*-values across treatments was as follows: Nitrite > HPE_300 > PPE_30 = PPE_300 > HPE_30 > Control.

The CIE b*-values also showed notable treatment effects (*p* < 0.001). Both HPE and PPE increased the b*-values, contributing to a more intense yellowness in the cooked products compared to the control group. A dose-dependent effect was observed for both treatments, with higher doses resulting in higher b*-values. Notably, HPE treatments consistently produced higher b*-values than PPE treatments at equivalent doses. Specifically, the addition of HPE at higher doses (300 mg TPC/kg) produced cooked products with increased yellowness, and the HPE_300 samples had the highest b*-values. In contrast, the PPE_30 and nitrite groups displayed the lowest b*-values. The CIE b* values decreased in the following order: HPE_300 > PPE_300 > HPE_30 > Control > PPE_30 > Nitrite.

**Figure 1:**
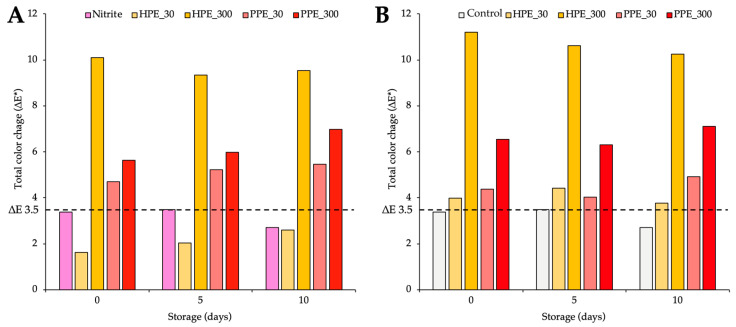
Total colour change (ΔE*) compared with the control group (**A**) and compared with the nitrite group (**B**) during storage. Bars above the dotted line at 3.5 indicate a clear perceptible difference in colour when comparing the products, with the control/nitrite groups showing distinguishable variations as perceived by the consumer.

Significant differences in the colour parameters, particularly in the a* and b* values, led to significant total colour differences (ΔE*), showing large variations compared to the control group due to the addition of nitrite or PPE/HPE (Figure 1).

Compared to the control (Figure 1A), products with 150 mg/kg sodium nitrite showed ΔE* changes below 3.5 throughout storage (2.71 to 3.49).

HPE and PPE showed a dose-dependent effect on ΔE* values, with higher concentrations leading to higher values. The addition of HPE exceeded ΔE* 3.5 only at 300 mg TPC/kg (9.3 to 10.1), while no change occurred at 30 mg TPC/kg (1.6 to 2.6). In contrast, the inclusion of PPE induced changes greater than 3.5 at both the 30 mg TPC/kg (4.7 to 5.5) and 300 mg TPC/kg (ranging from 5.6 to 7.0) doses.

Compared to the nitrite group (Figure 1B), HPE and PPE increased ΔE* values above 3.5, with HPE causing more noticeable changes (3.8 to 4.4 at 30 mg TPC/kg, 10.2 to 11.2 at 300 mg TPC/kg) than PPE (4.0 to 4.9 at 30 mg TPC/kg, 6.3 to 7.1 at 300 mg TPC/kg), with effects becoming more significant at higher extract levels.

### 3.3. Lipid Oxidation: Lipid Hydroperoxides (LOOH), Malondialdehyde (MDA), and Saturated Aldehyde and 4-Hydroxinonenal (4-HNE) Production

The primary and final oxidation products were significantly (*p* < 0.001) influenced by the addition of nitrite or PPE/HPE extracts (Table 4).

The formation of lipid hydroperoxides, an initial oxidation product, was significantly (*p* < 0.05) reduced in samples treated with nitrite, pomegranate peel extracts (PPE_30 and PPE_300), and prickly pear peel extract, particularly at higher concentrations (HPE_300), compared to the control group across all sampling times. This demonstrates enhanced oxidative stability. However, the addition of prickly pear peel extract at 30 mg TPC/kg (HPE_30) significantly (*p* < 0.05) increased LOOH levels, exceeding even those in the control samples. Over the storage period, LOOH content increased in the control, HPE (30 and 300 mg TPC/kg), and PPE_300 groups, while a contrasting trend was observed in cooked products formulated with nitrite and PPE_30, where LOOH levels declined, maintaining significant (*p* < 0.05) differences between the experimental groups.

The addition of nitrite, along with different concentrations of PPE and HPE, had a significant (*p* < 0.001) impact on MDA formation and accumulation. Nitrite effectively controlled MDA formation, resulting in significantly (*p* < 0.05) lower levels compared to the control and HPE groups. Similarly, peel extracts exhibited varying degrees of lipid oxidation inhibition, with PPE demonstrating greater efficacy than HPE. Nitrite and PPE, regardless of concentration, showed the highest suppression of MDA formation, maintaining levels below 3.0 µmol/g at all time points. No significant (*p* > 0.05) differences in MDA content were found between the nitrite and PPE groups, suggesting that PPE is as effective as nitrite in controlling lipid oxidation. However, a dose-dependent effect was observed only in the HPE groups, where MDA levels decreased as extract concentration increased.

Although HPE_300 reduced MDA formation, its effectiveness was significantly (*p* < 0.05) lower than that of PPE (ranging from 15.0 to 36.7 µmol/g for HPE_30 and ranging from 19.6 to 29.4 µmol/g for HPE_300, respectively). Notably, HPE at 30 mg TPC/kg in cooked products did not reduce lipid oxidation, as MDA levels remained comparable to those in the control group. Overall, MDA content significantly (*p* < 0.001) increased throughout the storage period in all experimental groups except for nitrite and PPE_300. In untreated controls, MDA levels rose significantly (*p* < 0.001) (from 19.1 to 32.9 µmol/g between day 0 and day 10).

Consistent with findings for LOOH and MDA, the concentrations of 4-hydroxynonenal and saturated aldehydes were significantly (*p* < 0.001) influenced by nitrite or peel extracts. The formation of saturated fatty aldehydes, such as hexanal, was significantly (*p* < 0.05) suppressed in products treated with nitrite or peel extracts. Overall, aldehydes followed a similar pattern in response to nitrite, HPE, or PPE, with storage time further increasing their concentrations. Neither nitrite nor PPE at doses of 30 and 300 mg TPC/kg, nor HPE at 300 mg TPC/kg, caused significant (*p* < 0.05) changes in 4-HNE content. In contrast, the inclusion of HPE at a lower dose (30 mg TPC/kg) led to a significant (*p* < 0.05) increase in 4-HNE levels, which were significantly (*p* < 0.05) higher than those in other groups at the end of storage (1.7; 1.0, 3.0, 1.7; 1.4 and 1.9 nmol/g for control, nitrite, HPE_30, HPE_300, PPE_30 and PPE_300, respectively). This trend aligns with previous observations for LOOH and MDA.

Hexanal (C6-al) content was significantly (*p* < 0.05) reduced by nitrite compared to the control group (10.2–75.9 vs. 1.0–0.0 nmol/g for control and nitrite, respectively). Notably, PPE, especially at the higher dose (300 mg TPC/kg), resulted in a significantly greater reduction in C6-al and other aldehyde concentrations, paralleling the effect observed in nitrite-treated samples, further supporting its strong inhibitory potential against lipid degradation. PPE_30 and PPE_300 samples had significantly (*p* < 0.05) lower C6-al levels (1.4–1.0 and 0.6–0.9 nmol/g, respectively) compared to the control. In contrast, the antioxidant effect of HPE was dose-dependent, with only the 300 mg TPC/kg dose significantly (*p* < 0.05) reducing hexanal formation compared to the control (0.5–9.9 nmol/g). The lower dose (30 mg TPC/kg) had no impact on hexanal formation (12.6–64.1 nmol/g), indicating that HPE, particularly at lower concentrations, was less effective in mitigating secondary oxidation products. Other aldehydes followed a similar trend to hexanal, with minor variations that were not further discussed due to their limited relevance.

### 3.4. Protein Oxidation: Carbonyls, Thiols, Tryptophan Fluorescence, Dityrosine, and Schiff Base Fluorescence Spectroscopy

The extent of protein oxidation in cooked samples over time was tracked by evaluating the carbonyl and thiol contents as well as tryptophan fluorescence, dityrosine, and Schiff base fluorescence spectroscopy (Table 5).

Overall, nitrite, HPE, and PPE significantly (*p* < 0.05) reduced the formation and accumulation of carbonyls compared to the control group, demonstrating their protective effects against protein carbonylation. Initially, nitrite addition led to a significant (*p* < 0.05) reduction in protein carbonyls (9.4 nmol/mg protein), whereas PPE showed no effect at any dose at day 0 (Control: 12.6; PPE_30: 12.3; and PPE_300: 10.8 nmol/mg protein). In contrast, HPE at 30 mg TPC/kg significantly (*p* < 0.05) increased protein carbonylation (31.4 nmol/mg protein).

During storage, carbonyl levels increased (*p* < 0.001) in the control group, as well as the nitrite and PPE_30 batches. However, in the HPE_30 and HPE_300 products, carbonyl contents were significantly (*p* < 0.01) reduced. The results indicate a dose-dependent effect of both HPE and PPE on carbonyl formation. Higher doses of HPE and PPE were associated with a significantly (*p* < 0.05) greater reduction in carbonyl content compared to lower doses. Notably, PPE exhibited a stronger protective effect against protein oxidation than HPE, leading to a more substantial reduction in carbonyl concentrations at higher inclusion levels throughout the storage period (HPE_30: 24.4; HPE_300: 18.4 vs. PPE_30: 16.4; PPE_300: 9.7 nmol/mg protein at day 10). These findings underscore the potential of PPE as a more effective agent than HPE in preserving protein against oxidative damage.

Immediately after the production of cooked products (day 0), no significant (*p* > 0.05) effects of nitrite, HPE, or PPE on thiol content were observed. However, notable changes in thiol amounts occurred during storage. Throughout the storage period, thiol content decreased in all groups except for PPE_300, which maintained stable content. Notably, nitrite addition resulted in a significant (*p* < 0.05) reduction in thiol content compared to the control group (40.7 vs. 35.8 nmol Cys eq./mg protein for control and nitrite at day 10), suggesting a pro-oxidative effect. PPE-treated samples exhibited significantly (*p* < 0.05) higher thiol concentrations than those in the control, nitrite, and HPE groups, with the effect being more pronounced at the higher inclusion level of 300 mg TPC/kg compared to 30 mg TPC/kg (40.7, 35.8, 38.3, 33.2, 42.1, and 46.2 nmol Cys eq./mg protein for control, nitrite, HPE_30, HPE_300, PPE_30, and PPE_300, respectively, at day 10). In contrast, HPE addition led to a reduction in thiol content relative to the control group. The loss of thiols was significantly (*p* < 0.05) greater in the HPE_300 group, which exhibited thiol levels that were substantially lower than the control and did not differ significantly (*p* > 0.05) from the nitrite group.

A significant (*p* < 0.05) decrease in tryptophan fluorescence intensity was observed in nitrite, HPE, and PPE products at 300 mg TPC/kg compared to the control group and peel extracts at 30 mg TPC/kg, indicating a dose-dependent effect in HPE and PPE. Storage had a negligible impact on tryptophan fluorescence.

HPE treatment significantly (*p* < 0.05) increased dityrosine content compared to the control, demonstrating a clear dose-dependent effect. Samples treated with HPE_300 exhibited significantly (*p* < 0.05) higher dityrosine levels than those treated with HPE_30, confirming the impact of dosage on dityrosine formation. Similarly, nitrite treatment led to a significant (*p* < 0.05) increase in dityrosine content compared to the control group. In contrast, PPE treatment did not alter dityrosine content, as no significant (*p* > 0.05) differences were observed between the control and PPE-treated samples, regardless of the dose. Similarly, storage had a minor effect on dityrosine levels.

Similarly, Schiff base content was significantly (*p* < 0.05) influenced by treatment with HPE, whereas storage alone did not alter Schiff base levels. In HPE samples, a clear dose-dependent effect (*p* < 0.05) was noticed, with a higher dose leading to a greater increase in Schiff base content. Notably, the highest Schiff base content was recorded at the highest HPE dose (HPE_300). Both HPE_30 and HPE_300 showed significant (*p* < 0.05) differences compared to the control, nitrite, and PPE_30 groups. These latter groups did not exhibit significant (*p* > 0.05) differences among themselves. Interestingly, the Schiff base content in the PPE_300 group was not significantly (*p* > 0.05) different from that in the HPE_30 group, suggesting that while HPE had a strong dose-dependent effect, PPE at higher doses did not elicit a comparable response.

**Figure 2:**
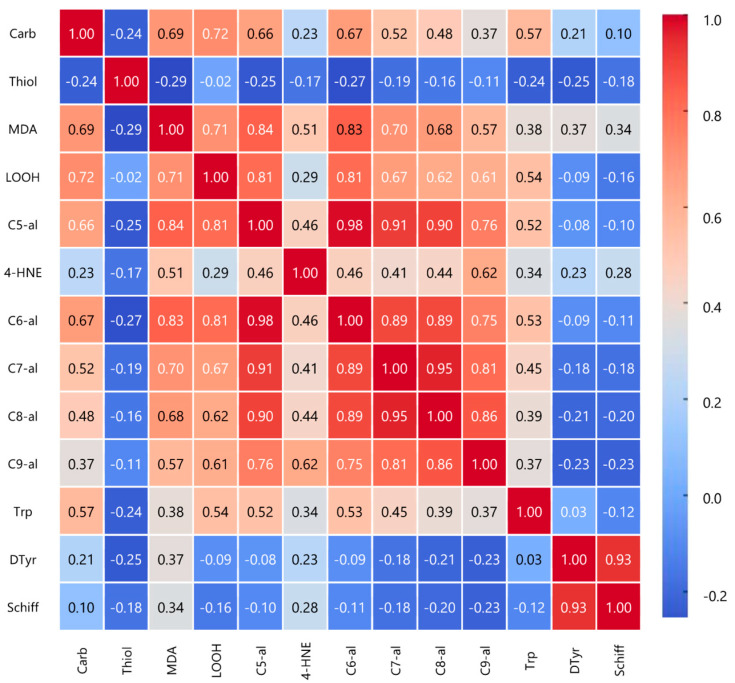
Pearson correlation heatmap. It visually represents the relationships between different oxidative stress markers, highlighting strong positive and negative correlations. (Carb: carbonyls; Thiol: thiols; MDA: malondialdehyde; LOOH: lipid hydroperoxides; C5-al: pentanal; 4-HNE: 4-hidroxynonenal; C6-al: hexanal; C7-al: heptanal; C8-al: octanal; C9-al: nonenal; Trp: tryptophan; DTyr: ditiroxine, Schiff: Schiff bases).

The Pearson correlation analysis reveals significant associations among oxidative stress markers (Figure 2). Carbonyls positively correlate with malondialdehyde (r = 0.690, *p* < 0.01) and lipid hydroperoxides (r = 0.720, *p* < 0.01), suggesting a potential role of lipid peroxidation in protein oxidation. Notably, MDA strongly correlates with C5-al (r = 0.837, *p* < 0.01), C6-al (r = 0.835, *p* < 0.01), and C7-al (r = 0.701, *p* < 0.01), indicating a shared pathway in aldehyde formation. LOOH shows strong correlations with C5-al (r = 0.805, *p* < 0.01) and C6-al (r = 0.808, *p* < 0.01). Interestingly, thiols negatively correlate with MDA (r = −0.291, *p* < 0.01) and C5-al (r = −0.252, *p* < 0.05), consistent with their protective role against oxidative damage. Among aldehydes, C5-al to C9-al demonstrate strong intercorrelations (e.g., C5-al vs. C6-al, r = 0.984, *p* < 0.01), supporting a common oxidation mechanism. Additionally, 4-HNE plays a central role in oxidative stress, correlating positively with both lipid and protein oxidation markers. It is strongly associated with primary (LOOH, r = 0.290, *p* < 0.01) and end products of lipid peroxidation (MDA, r = 0.506, *p* < 0.01), as well as aldehydes (C5-al to C7-al). Furthermore, 4-HNE correlates with protein oxidation markers, including tryptophan fluorescence intensity (r = 0.338, *p* < 0.01), dityrosine (r = 0.228, *p* < 0.05), and Schiff bases (r = 0.277, *p* < 0.01), indicating its involvement in protein modifications.

## 4. Discussion

The present study aimed to evaluate the antioxidative and preservative effects of two fruit peel extracts (prickly pear and pomegranate) with different antioxidant and pigment content in cooked chicken products, focusing on instrumental colour coordinates, lipid and protein oxidation, and overall oxidative stability during storage.

Chemical analysis of the normalized HPE and PPE extracts at 30 and 300 mg TPC/mL revealed differences in their phenolic composition and antioxidant activities. Notably, certain compounds, such as condensed tannins, were only detected in PPE at the higher concentration (PPE_300), likely due to their presence in low amounts that fall below detection thresholds at lower concentrations. This suggests a more diverse and complex polyphenolic profile in PPE. Antioxidant assays further confirmed the superior activity of PPE compared to HPE, particularly at higher concentrations. In the FRAP assay, PPE_300 exhibited significantly greater reducing power than HPE_300. Similarly, dose-dependent increases in DPPH and ABTS radical scavenging activities were observed in both extracts, with PPE consistently outperforming HPE. These differences in antioxidant activity, despite equal total phenolic content, are likely attributed to a qualitative difference in the phenolic compound composition between the two extracts [13,22]. PPE exhibited greater antioxidant activity, highlighting its potential as a potent natural antioxidant, as previously reported [16], and as will be discussed further.

The addition of PPE or HPE significantly influenced the instrumental CIE L*a*b* colour parameters measured under D65 illumination compared to both the control and nitrite-treated groups. These changes can be primarily attributed to the unique coloured compounds present in each extract and their respective concentrations. Among the colour parameters, the b*-value (yellowness) shows the most noticeable variation, indicating that the inclusion of either HPE or PPE predominantly impacts this aspect of the cooked product colour. The observed differences arise from the specific pigments’ characteristic of each extract. Due to the colour characteristics of the experimental samples, our study suggests that carotenoids may be more prevalent in PPE, while indicaxanthin could be more abundant in HPE, contributing to increased yellowness [11,23]. These findings are consistent with previous research, which has reported similar effects of pomegranate and prickly pear on the colour properties of different meat products [9,16,21].

Importantly, both extracts induced a noticeable ΔE* compared to the control or nitrite groups, with PPE demonstrating a more moderate colour change compared to HPE, suggesting that the choice of extract and its concentration should be optimized to balance antioxidative effects with colour attributes. The observed changes in ΔE* in samples formulated with PPE or HPE suggest visual modifications that could influence consumer acceptability. Variations in ΔE* throughout storage were largely driven by a significant decrease in the CIE a*-value, signifying a transition from red toward green coloration; this change likely reflects alterations in pigment composition or degradation processes occurring over time.

These findings are consistent with previous studies, which reported significant changes in ΔE* following the removal of nitrite or the addition of PPE or HPE to chicken cooked product formulations [9,16,18,19,21]. Notably, a ΔE* value greater than 3.5 indicates a perceptible difference in colour [37]. In both cases, the inclusion of either extract results in perceptible colour changes, with HPE exhibiting a much more substantial impact compared to PPE.

The well-known antioxidant activity of nitrites plays a crucial role in efficiently controlling the formation of lipid primary oxidation products, such as LOOH, as well as final products like MDA and saturated aldehydes as well as carbonyls from protein oxidation [2,3].

In samples without nitrite, the formation and accumulation of primary (LOOH) and secondary lipid oxidation products (MDA and fatty aldehydes) are closely associated with the antioxidant activity of pomegranate and prickly pear peel extracts. Our results indicate that, at both tested concentrations, PPE extracts demonstrated significantly greater control over the formation of these compounds compared to HPE extract counterparts. This finding suggests that the antioxidant capacity of the extracts cannot be fully explained by the normalized total phenolic content. Although both extracts may contain similar concentrations of phenolics, their antioxidant effectiveness varies considerably, as reported for ABTS, FRAP, and DPPH activities (Table 2). This implies that PPE extracts contain certain compounds that are more effective mechanisms against free radicals, acting as electron donors, quenchers, and reducing agents, resulting in greater protection against lipid oxidation at any of the assayed doses. The dose-dependent effect of HPE indicates that the antioxidant efficacy of prickly pear peel extract is highly concentration-dependent. In contrast, PPE maintained consistent antioxidant performance regardless of dosage. Results show that PPE at a low dose was effective in controlling oxidation, whereas HPE required a higher dose to achieve similar effects. Under these conditions, PPE proved to be even more effective than HPE. This outcome underscores the potential of PPE as an effective alternative to nitrite.

Notably, the inhibition of lipid oxidation by pomegranate extracts, irrespective of the dose, and by prickly pear extracts at the highest dose, is consistent with previous findings reported across a range of meat products, thereby confirming their antioxidant efficacy regardless of product type and dose [9,11,16,18,19,21,24]. The generation of oxidation end-products, MDA and hexanal from n-6 fatty acids during lipid oxidation, is closely linked to rancidity, as these compounds are markers of oxidative degradation, and their accumulation can have significant implications for health, including increased risk of chronic diseases due to their potential as reactive aldehydes that can cause cellular damage [38].

Protein oxidation, as evidenced by carbonyl and thiol content analysis, was significantly mitigated by nitrite and PPE even at a lower dose. In contrast, HPE, particularly at 30 mg TPC/kg, failed to prevent protein oxidation. The balance between the antioxidants in the extracts and the lipid oxidation products generated helps to promote or control protein oxidation It is known that lipid oxidation products can readily react with certain amino acids in proteins to form carbonyls [39]. Therefore, the higher lipid oxidation found in the HPE batch with 30 mg TPC/kg, together with the lower efficacy of its antioxidant molecules, promotes a higher development of protein carbonylation processes. Our study aligns with previous research, which has shown that pomegranate juice and its by-products can prevent protein oxidation and reduce carbonyl accumulation in muscle foods. Notable studies, such as Horbańczuk et al. [40], Fourati et al. [41], Smaoui et al. [42], and Turgut et al. [43,44], found that pomegranate peel extract reduced carbonyl levels in beef, minced products, and Cava and Ladero in dry sausage [21]. To the best of our knowledge, there are no published scientific studies on protein oxidation in relation to prickly pear.

Our findings suggest that while PPE effectively preserves thiol content, particularly at higher concentrations, nitrite and HPE may promote thiol loss, potentially accelerating protein oxidation during storage. The level of free thiols in meat products results from a balance between -SH group oxidation, nitrosation by nitrite, and the formation of protein-phenolic adducts via quinone binding [45,46,47]. The stability of thiols in the PPE_300 group highlights its superior protective effect against oxidative degradation, further supporting its potential as an effective antioxidant in cooked products.

During storage, thiol loss occurs regardless of treatment, but the addition of PPE does not increase thiol depletion. In nitrite-treated sausages, thiol losses may be linked to nitrosation, while HPE treatment also reduces thiol levels. Although both nitrite and HPE likely involve cysteine oxidation, distinct mechanisms might be responsible for thiol loss in cured and HPE-added products. Previous findings suggest that nitrosylcysteine formation could explain the reduced thiol levels in nitrite-treated samples, while in HPE-added sausages, cysteine-polyphenolic compound adducts may contribute to thiol loss, particularly with increasing HPE doses [21].

As previously reported, PPE effectively preserves protein integrity by maintaining tryptophan fluorescence and reducing dityrosine and Schiff base formation, indicating strong antioxidative capacity. Unlike HPE, which at high doses unexpectedly decreases tryptophan fluorescence and increases dityrosine and Schiff bases, PPE consistently outperforms HPE in lipid oxidation inhibition and protein protection. The interdependent relationships between lipid and protein oxidation are evidenced by correlations between lipid and protein oxidative markers shown in Figure 2. Additionally, antioxidants from extracts play a crucial role in preventing both types of oxidation by neutralizing free radicals and reactive species, thus protecting lipids and proteins from oxidative damage [5].

## 5. Conclusions

The findings of this study highlight the potential of plant-based antioxidants for food preservation. Specifically, natural extracts—particularly pomegranate peel extract—demonstrated promising antioxidant activity in meat systems, showing efficacy comparable to nitrite in preserving lipid and protein stability when applied at 300 mg total phenolic compounds/kg. Among the tested treatments, PPE_300 exhibited the strongest antioxidant effects, surpassing HPE_300, and emerging as a candidate for reducing the reliance on synthetic additives.

However, while PPE shows potential, its suitability for industrial application requires further validation. Critical parameters such as sensory quality, consumer acceptability, microbial stability, and toxicological safety need to be thoroughly investigated to support claims regarding its practical use in food products.

Additionally, the current study did not evaluate batch-to-batch consistency or the stability of the extract over time—important considerations for industrial scalability. Future research should address these limitations and focus on optimizing PPE or HPE incorporation methods across a range of meat matrices and storage conditions. Exploring extract interactions with other natural antioxidants and refining extraction processes for enhanced bioactive retention may further improve its effectiveness.

Ultimately, this study contributes to the development of clean-label preservation strategies and aligns with consumer trends favouring natural alternatives.

## Figures and Tables

**Table 1:** Formulation of the cooked chicken models.

Component	0_Nitr	150_Nitr	30_PPE	300_PPE	30_HPE	300_HPE
Chicken (g/kg)	780	780	780	780	780	780
NaCl (g/kg)	20	20	20	20	20	20
NaNO_2_ (g/kg)	0	0.15	0.0	0.0	0.0	0.0
Peel extract (mL) *	0	0.0	0.3	3.1	0.3	3.1
Distilled water (g/kg)	200	200	199.7	196.9	199.7	196.9

* Peel extract from PPE or HPE to provide 30 or 300 mg total phenolic compounds/kg.

**Table 2:** Characterization of HPE and PPE extracts.

	Prickly Pear Extract		Pomegranate Peel Extract	
	HPE_30	HPE_300	PPE_30	PPE_300
Total phenolics compounds (µg/mL)	31.5 b ± 0.27	320.6 a ± 12.14	31.5 b ± 0.53	328.9 a ± 3.22
Condensed tannins (µg/mL)	n.d.	n.d.	n.d.	1.2 ± 0.14
FRAP (µg Fe^2+^/mL)	15.4 d ± 1.52	150.1 b ± 3.03	54.8 c ± 1.35	555.8 a ± 13.16
DPPH (mg Trolox eq./mL)	8.3 c ± 0.78	73.7 b ± 7.40	68.2 b ± 1.53	627.5 a ± 51.75
ABTS (mg Trolox eq./mL)	44.8 c ± 4.06	501.9 b ± 34.44	80.8 c ± 4.42	807.8 a ± 34.22

a,b,c,d: Means within the same parameter with different letters are significantly different (Tukey’s test, *p* < 0.05). n.d.: not detected.

**Table 3:** Instrumental colour coordinates in cooked chicken model during refrigerated storage.

			Prickly Pear Peel Extract		Pomegranate Peel Extract		
Day	Control	Nitrite	HPE_30	HPE_300	PPE_30	PPE_300	Sig.
L*							
0	77.8 a y ± 0.27	77.1 a ± 0.23	77.3 a ± 0.62	74.2 b ± 0.44	73.3 b ± 0.56	73.3 b x ± 0.88	***
5	78.7 a x ± 0.41	77.5 a ± 0.47	77.6 a ± 0.9	74.1 b ± 0.38	73.9 b ± 0.84	73.3 b x ± 0.44	***
10	78.2 a xy ± 0.63	77.6 ab ± 0.51	76.6 b ± 0.94	73.9 c ± 0.8	72.9 cd ± 0.42	72.1 d y ± 0.42	***
Sig.	*	n.s.	n.s.	n.s.	n.s.	*	
a*							
0	0.3 e ± 0.18	3.2 a x ± 0.21	0.7 d ± 0.21	2.4 b x ± 0.14	1.1 c ± 0.08	1.1 c ± 0.16	***
5	0.3 d ± 0.2	2.9 a y ± 0.15	0.5 d ± 0.18	1.7 b y ± 0.17	1.1 c ± 0.12	1.2 c ± 0.06	***
10	0.0 e ± 0.13	2.4 a z ± 0.06	0.5 d ± 0.24	1.4 b z ± 0.09	1.0 c ± 0.08	1.1 c ± 0.11	***
Sig.	n.s.	***	n.s.	***	n.s.	n.s.	
b*							
0	11.8 d y ± 0.26	10.2 f ± 0.17	13.3 c y ± 0.07	21.0 a ± 0.41	10.7 e y ± 0.18	15.1 b y ± 0.2	***
5	12.7 d x ± 0.25	10.7 e ± 0.14	14.4 c x ± 0.47	20.7 a ± 0.21	10.8 e y ± 0.25	15.1 b y ± 0.16	***
10	12.3 d xy ± 0.64	11.2 e ± 0.16	14.3 c x ± 0.7	20.7 a ± 0.1	11.5 e x ± 0.12	15.5 b x ± 0.29	***
Sig.	n.s.	***	**	n.s.	***	*	

Sig.: significance; n.s.: not significant (*p* > 0.05); *: *p* < 0.05; **: *p* < 0.01; ***: *p* < 0.001. a,b,c,d,e,f: Means within the same time of sampling with different letters are significantly different (Tukey’s test, *p* < 0.05). x,y,z: Means within the same experimental group with different letters are significantly different (Tukey’s test, *p* < 0.05).

**Table 4:** Evolution of lipid oxidation markers in the cooked chicken model during refrigerated storage.

			Prickly Pear Extract		Pomegranate Peel Extract		
Day	Control	Nitrite	HPE_30	HPE_300	PPE_30	PPE_300	Sig.
Lipid hydroperoxides (nmol/g)							
0	0.8 b z ± 0.07	0.2 c x ± 0.19	2.7 a ± 0.28	0.0 c y ± 0.000	0.1c y ± 0.17	0.0 c y ± 0.09	***
5	1.9 b y ± 0.22	0.0 d y ± 0.00	3.3 a ± 0.54	0.0 d y ± 0.02	0.3 cd x ± 0.13	0.7 c x ± 0.08	***
10	2.5 b x ± 0.05	0.0 d xy ± 0.07	3.1 a ± 0.30	0.2 d x ± 0.02	0.1 d y ± 0.09	0.8 c x ± 0.3	***
Sig.	***	*	n.s.	***	*	***	
Malondialdehyde (µmol/g)							
0	19.1 a y ± 1.17	3.3 c ± 0.66	15.0 b y ± 2.23	19.6 a z ± 1.45	1.7 c y ± 0.34	2.8 c ± 0.19	***
5	31.7 a x ± 3.67	2.8 c ± 0.28	36.0 a x ± 5.11	23.7 b y ± 1.34	2.4 c x ± 0.34	3.0 c ± 0.36	***
10	32.9 ab x ± 1.6	2.6 c ± 0.33	36.7 a x ± 8.82	26.4 b x ± 1.13	2.7 c x ± 0.44	2.5 c ± 0.40	***
Sig.	***	n.s.	***	***	**	n.s.	
4-HNE (nmol/g)							
0	1.0 b z ± 0.24	1.5 ab ± 0.20	1.0 b y ± 0.39	2.3 a ± 0.27	1.7 ab ± 0.08	1.0 b ± 0.89	***
5	2.5 a x ± 0.15	1.2 bc ± 0.81	1.9 abc y ± 0.79	2.1 ab ± 0.25	1.4 abc ± 0.12	0.9 c ± 0.66	***
10	1.7 b y ± 0.41	1.0 b ± 0.14	3.0 a x ± 0.67	1.7 b ± 0.77	1.4 b ± 0.79	1.9 b ± 0.09	***
Sig.	***	n.s.	***	n.s.	n.s.	n.s.	
Pentanal (nmol/g)							
0	3.0 a y ± 0.68	0.8 b ± 0.13	3.5 a y ± 0.74	0.5 b z ± 0.12	0.9 b ± 0.13	0.6 b ± 0.31	***
5	14.1 a x ± 2.45	0.6 c ± 0.27	9.9 b x ± 2.75	1.8 c y ± 0.31	0.7 c ± 0.09	0.9 c ± 0.20	***
10	11.6 a x ± 0.43	0.5 b ± 0.05	11.1 a x ± 4.66	2.6 b x ± 0.07	0.9 b ± 0.21	1.0 b ± 0.58	***
Sig.	***	n.s.	**	***	n.s.	n.s.	
Hexanal (nmol/g)							
0	10.2 a y ± 2.56	1.0 b x ± 0.18	12.6 a y ± 2.57	0.5 b z ± 0.10	1.4 b x ± 0.22	0.6 b ± 0.44	***
5	71.0 a x ± 10.98	0.6 c y ± 0.24	55.0 b x ± 14.24	6.3 c y ± 1.22	0.9 c y ± 0.18	0.7 c ± 0.22	***
10	75.9 a x ± 4.98	0.6 b y ± 0.69	64.1 a x ± 25.69	9.9 b x ± 0.46	1.0 b y ± 0.17	0.9 b ± 0.11	***
Sig.	***	**	***	***	**	n.s.	
Heptanal (nmol/g)							
0	0.7 a y ± 0.24	0.3 bc x ± 0.164	0.3 bc y ± 37.0	0.1 c y ± 0.1	0.5 ab x ± 0.16	0.2 c ± 0.21	***
5	3.0 a x ± 0.75	0.0 c y ± 0.04	1.6 b x ± 0.427	0.3 c xy ± 0.19	0.3 c y ± 0.14	0.3 c ± 0.13	***
10	2.1 a x ± 1.14	0.0 c y ± 0.00	1.8 a x ± 0.793	0.3 b x ± 0.11	0.4 b xy ± 0.14	0.4 b ± 0.08	***
Sig.	**	***	**	*	*	n.s.	
Octanal (nmol/g)							
0	0.7 a z ± 0.17	0.4 bc x ± 0.11	0.4 bc y ± 0.05	0.2 c y ± 0.03	0.6 ab x ± 0.15	0.3 c ± 0.23	***
5	2.4 a x ± 0.48	0.2 c xy ± 0.15	1.1 b x ± 0.21	0.4 c x ± 0.12	0.3 c y ± 0.12	0.3 c ± 0.13	***
10	1.6 a y ± 0.36	0.1 b y ± 0.06	1.2 a x ± 0.51	0.4 b x ± 0.06	0.5 b xy ± 0.09	0.4 b ± 0.04	***
Sig.	***	**	**	***	**	n.s.	
Nonanal (nmol/g)							
0	0.3 z ± 0.94	0.3 x ± 0.06	0.2 y ± 0.09	0.1 ± 0.03	0.3 ± 0.05	0.2 ± 0.00	n.s.
5	1.0 a x ± 0.18	0.1 c xy ± 0.20	0.6 b x ± 0.13	0.2 c ± 0.05	0.3 bc ± 0.16	0.5 c ± 0.00	***
10	0.6 ab y ± 0.09	0.1 c y ± 0.03	0.8 a x ± 0.34	0.2 c ± 0.12	0.2 c ± 0.15	0.4 bc ± 0.00	***
Sig.	***	n.s.	**	n.s.	n.s.	n.s.	

Sig.: significance; n.s.: not significant (*p* > 0.05); *: *p* < 0.05; **: *p* < 0.01; ***: *p* < 0.001. a,b,c: Means within the same time of sampling with different letters are significantly different (Tukey’s test, *p* < 0.05). x,y,z: Means within the same experimental group with different letters are significantly different (Tukey’s test, *p* < 0.05).

**Table 5:** Evolution of protein oxidation markers in the cooked chicken model during refrigerated storage.

			Prickly Pear Peel Extract		Pomegranate Peel Extract		
Day	Control	Nitrite	HPE_30	HPE_300	PPE_30	PPE_300	Sig.
Protein Carbonyl Compounds (nmol/mg protein)							
0	12.6 c y ± 2.15	9.4 d z ± 1.21	31.4 a x ± 2.36	20.9 b x ± 1.31	12.3 cd y ± 0.77	10.8 cd ± 1.09	***
5	25.0 a x ± 3.93	16.8 b y ± 2.91	28.9 a x ± 1.13	17.9 b y ± 1.99	16.6 b x ± 1.41	10.0 c ± 1.21	***
10	30.7 a x ± 4.46	20.4 bc x ± 1.70	24.4 b y ± 1.46	18.4 c xy ± 1.52	16.4 c x ± 0.78	9.7 d ± 1.22	***
Sig.	***	***	***	*	***	n.s.	
Tryptophan fluorescence (RFU)							
0	397 c y ± 185.0	544 abc ± 32.4	647 a ± 53.9	486 bc ± 31.3	623 ab ± 35.0	410 c ± 24.7	***
5	620 a x ± 25.3	528 b ± 27.1	656 a ± 50.4	511 b ± 34.8	620 a ± 40.2	432 c ± 44.8	***
10	669 ab x ± 56.8	515 cd ± 28.0	722 a ± 75.9	520 cd ± 35.5	604 bc ± 45.0	461 d ± 70.8	***
Sig.	**	n.s.	n.s.	n.s.	n.s.	n.s.	
Dityrosine (RFU)							
0	2.1 c ± 0.70	2.6 bc ± 0.17	3.0 b ± 0.41	4.1 a y ± 0.08	2.1 c y ± 0.09	1.9 c y ± 0.10	***
5	2.1 c ± 0.15	2.5 bc ± 0.12	2.7 b ± 0.30	4.6 a x ± 0.23	2.3 c x ± 0.12	2.3 c x ± 0.14	***
10	2.1 c ± 0.21	2.5 b ± 0.03	2.9 b ± 0.35	4.1 a y ± 0.15	1.9 c y ± 0.11	2.0 c y ± 0.11	***
Sig.	n.s.	n.s.	n.s.	***	**	***	
Schiff base fluorescence spectroscopy (RFU)							
0	0.4 c ± 0.14	0.4 c ± 0.02	0.7 b ± 0.13	1.4 a ± 0.06	0.4 c ± 0.04	0.5 bc y ± 0.03	***
5	0.4 c ± 0.04	0.4 c ± 0.01	0.6 b ± 0.04	1.5 a ± 0.15	0.5 c ± 0.02	0.7 b x ± 0.05	***
10	0.5 cd ± 0.04	0.4 d ± 0.03	0.7 b ± 0.15	1.4 a ± 0.09	0.4 cd ± 0.03	0.6 bc xy ± 0.07	***
Sig.	n.s.	n.s.	n.s.	n.s.	n.s.	*	
Thiols (nmol Cys eq./mg protein)							
0	49.6 x ± 3.83	48.4 x ± 2.53	51.1 x ± 2.28	51.2 x ± 1.42	51.3 x ± 2.75	46.5 ± 2.87	n.s.
5	39.5 bc y ± 2.19	35.4 c y ± 0.57	41.4 b y ± 4.76	36.6 bc y ± 2.10	40.8 b y ± 2.05	47.1 a ± 1.24	***
10	40.7 b y ± 2.83	35.8 cd y ± 1.51	38.3 bc y ± 3.40	33.2 d z ± 1.92	42.1 ab y ± 1.72	46.2 a ± 1.05	***
Sig.	***	***	***	***	***	n.s.	

Sig.: significance; n.s.: not significant (*p* > 0.05); *: *p* < 0.05; **: *p* < 0.01; ***: *p* < 0.001. a,b,c,d: Means within the same time of sampling with different letters are significantly different (Tukey’s test, *p* < 0.05). x,y,z: Means within the same experimental group with different letters are significantly different (Tukey’s test, *p* < 0.05).

## Data Availability

The data that support the findings of this study are openly available in Mendeley Data at 10.17632/bw7jvhhd35.1.

## Abbreviations

The following abbreviations are used in this manuscript:

4-HNE	4-Hydroxy-nonenal
C6-al	Hexanal
Cys eq.	Cysteine equivalents
HPE	Prickly pear peel extract
HPLC-FD	High performance liquid chromatography-fluorescence detector
LOOH	Lipid hydroperoxide
MDA	Malondialdehyde
PPE	Pomegranate peel extract
TPC	Total phenolic compounds
